# Coagulation Proteins Influencing Global Coagulation Assays in Cirrhosis: Hypercoagulability in Cirrhosis Assessed by Thrombomodulin-Induced Thrombin Generation Assay

**DOI:** 10.1155/2013/856754

**Published:** 2013-02-21

**Authors:** Nam Youngwon, Ji-Eun Kim, Hae Sook Lim, Kyou-Sup Han, Hyun Kyung Kim

**Affiliations:** ^1^Department of Laboratory Medicine, Seoul National University College of Medicine, 101 Daehak-ro, Jongno-gu, Seoul 110-744, Republic of Korea; ^2^Cancer Research Institute, Seoul National University College of Medicine, 101 Daehak-ro, Jongno-gu, Seoul 110-744, Republic of Korea

## Abstract

*Background*. Liver disease is accompanied by profound hemostatic disturbances. We investigated the influences of pro- and anticoagulation factors on global coagulation tests including prothrombin time (PT), activated partial thromboplastin time (aPTT), and thrombin generation assay (TGA) in cirrhosis. We also investigated whether cirrhotic patients exhibit hypo- or hypercoagulability using the TGA. *Methods*. The TGA was performed on a calibrated automated thrombogram, given lag time, endogenous thrombin potential (ETP), and peak thrombin in 156 cirrhotic patients and 73 controls. *Results*. PT was determined according to the factor (F) II, FV, FVII, FIX, and protein C levels. We observed that aPTT was dependent on FII, FIX, and FX levels. The ETP was dependent on FII, antithrombin, and protein C with 5 pM tissue factor (TF) stimulation, and FIX and protein C at 1 pM TF. The ETP ratio with 1 pM TF increased significantly in cirrhosis, indicating hypercoagulability, whereas that with 5 pM TF did not increase in cirrhosis. *Conclusion*. PT and the TGA are sensitive to protein C levels. Even with prolonged PT, the TGA can detect hypercoagulability in cirrhosis. Further studies should evaluate global coagulation status in cirrhosis patients using the newly devised TGA system.

## 1. Introduction

Prothrombin time (PT) and activated partial thromboplastin time (aPTT) are widely used in clinical laboratories as routine screening tests of the coagulation system. However, these tests cannot accurately predict hemorrhagic risk and vary among individuals [1]. Recently, there has been increasing evidence that thrombin generation provides useful information on the coagulation status. The thrombin generation assay (TGA) is a global coagulation test that measures the total amount of thrombin production triggered by tissue factor (TF) in plasma using an automated calibrated thrombogram. The resultant thrombogram has been validated as a good indicator of thrombotic and hemorrhagic conditions [2, 3]. 

Liver disease is accompanied by profound disturbances in the hemostatic system due to reduced plasma levels of pro- and anticoagulation factors synthesized by the liver [4]. Thus, the global effect of liver disease on hemostasis is very complex in that patients with advanced liver disease can experience bleeding or even thrombosis. In clinical practice, cirrhosis is generally accompanied by prolonged PT and aPTT due to impaired synthesis of most coagulation factors. Therefore, PT is widely used to predict bleeding risk in cirrhosis. However, despite PT prolongation, patients with cirrhosis seldom exhibit clinical bleeding events; hence, the PT may not accurately reflect the global hemostatic activity affected by various decreased levels of pro- and anticoagulation factors in cirrhosis. Several recent reports suggest that cirrhotic patients exhibit normal intact thrombograms as a result of acquired protein C deficiency despite prolonged PT [5–7]. However, the effect of protein C deficiency in cirrhosis remains unclear. Moreover, no existing report discusses how the individual levels of pro- and anticoagulant factors influence global coagulation test results in cirrhotic patients, including PT, aPTT, and TGA. 

In this study, we have investigated the influence of coagulation and anticoagulation factors on 3 global coagulation tests, that is, PT, aPTT, and TGA, in a population of patients with cirrhosis. We also investigated whether patients with cirrhosis exhibit hypo- or hypercoagulability using TGA stimulated by 2 different concentrations of TF. 

## 2. Materials and Methods

### 2.1. Study Population

In total, 156 adult patients with cirrhosis were included in the present study. Cirrhosis was diagnosed based on the clinical, laboratory, or radiologic findings. Exclusion criteria were inherited bleeding or thrombotic disorders, warfarin or heparin use within 7 days of blood collection, or body weight < 30 kg. The severity of cirrhosis was estimated according to the model for end-stage liver disease (MELD) score. The MELD score was used for evaluation of cirrhosis severity and calculated as follows: MELD score −10[0.957ln(creatinine, mg/dL) + 0.378ln(bilirubin, mg/dL) + 1.12ln(INR) + 0.643]. Peripheral venous blood samples were collected in commercially available tubes containing 0.109 M sodium citrate (Becton Dickinson, San Jose, CA, USA). The plasma was separated by centrifuging whole blood at 1550 ×g for 15 min within 2 h of blood collection. The aliquots of plasma were stored at −80°C. 

Seventy-three healthy adults for whom the coagulation screening tests were requested in routine health checkups were included as controls. The study protocol was reviewed and approved by the Institutional Review Board of Seoul National University College of Medicine. 

### 2.2. Thrombin Generation Assay

Thrombin generation in TF-triggered platelet-poor plasma (PPP) was measured by the calibrated automated thrombogram method (Thrombinoscope BV, Maastricht, The Netherlands) as described previously [8]. Briefly, 20 *μ*L reagent containing TF with final concentration of 5 or 1 pM (PPP reagent 5 pM and PPP reagent low, resp.; Thrombinoscope BV) and phospholipids or thrombin calibrators were dispensed into each well of round-bottom 96-well plates, and 80 *μ*L test plasma was added. In the same batch run, thrombomodulin (TM; Haematologic Technologies, Essex Junction, VT, USA) was added to the test plasma. The final concentration of TM was 5 nM for 5 pM TF stimulation and 2.5 nM for 1 pM TF stimulation. After the addition of 20 *μ*L fluorogenic substrate in hydroxyethyl-piperazineethanesulfonic acid (HEPES) buffer with CaCl_2_, the fluorescent signal was read in a Fluoroskan Ascent fluorometer (Thermo Labsystems OY, Helsinki, Finland). Thrombin generation curves were subsequently calculated with Thrombinoscope software version 3.0.0.29 (Thrombinoscope BV) and analyzed using parameters that describe the initiation, propagation, and termination phases of thrombin generation, namely, the lag time, endogenous thrombin potential (ETP), and peak thrombin concentration (peak thrombin). The lag time is equivalent to the clotting time and was defined as the time required to reach one-sixth of the peak height, which is the measure of the initiation phase. The peak height was defined as the maximum thrombin concentration. ETP is the area under the thrombin generation curve and represents the total amount of thrombin generated. The ETP ratio was calculated by dividing “ETP with TM” by “ETP without TM” multiplied by 100. Because TM is an anticoagulant, TM addition triggers the reduction of ETP value. Therefore, the ETP ratio reflects increased resistance to the anticoagulant action of TM. In other words, the increased ETP ratio represents hypercoagulability that resists anticoagulant activity of TM. In each plate containing an aliquot of control plasma (POOL NORM, Diagnostica Stago, France), the interassay and intra-assay coefficients of variation (CV) of ETP were 2.6% and 5.2%, respectively. 

### 2.3. Conventional Coagulation Tests

Coagulation tests including PT, aPTT, and factor assays were performed on an automated coagulation analyzer (ACL TOP, Beckman Coulter, Fullerton, CA, USA). PT, aPTT, and fibrinogen were measured with clotting method by using HemosIL RecombiPlasTin, SynthASil, and HemosIL Fibrinogen-C XL reagents, respectively (Instrumentation Laboratory SpA, Milan, Italy). Coagulation factors were assayed by a PT-based clotting assay using HemosIL RecombiPlasTin reagent for factor (F) II, FV, FVII, and FX as well as an aPTT-based clotting assay using SynthASil reagent for FVIII and FIX. Antithrombin and protein C levels were determined by chromogenic assays (HemosIL liquid antithrombin and HemosIL Protein C, resp.; Instrumentation Laboratory SpA), and protein S activity was measured by clotting assay (HemosIL Free Protein S, Instrumentation Laboratory SpA).

### 2.4. Statistical Analysis

Continuous variables were compared using the Mann-Whitney *U*  test and Kruskal-Wallis analysis. Meanwhile, categorical variables were compared using the *χ* ^2^  test. Correlations are expressed as Pearson's coefficients. Multiple linear regression analysis was performed to assess the relative effects of coagulation and anticoagulation factors on thrombin generation, PT, and aPTT. The adjusted *R* ^2^ and standardized regression coefficients (*β*) of the independent variables were calculated for each model. All analyses were carried out using SPSS version 12.0 (SPSS Inc., Chicago, IL, USA). The level of significance was set at  *P* < 0.05. 

## 3. Results

### 3.1. Effects of Pro- and Anticoagulation Factors on Global Coagulation Tests

The clinical and laboratory characteristics of the controls and patients are shown in Table 1. There were no significant differences between the controls and patients with respect to age, gender, WBC, or creatinine. Compared with the controls, PT and aPTT were significantly prolonged in the patients with cirrhosis (Table 1). Similarly, anticoagulation factors, including antithrombin, protein C, and protein S, were lower in the cirrhosis patients.

In the multiple linear regression analysis using data of cirrhotic patients (Table 2), the *β* value of FII for PT as a dependent variable was −0.413; this means that when FII increases by 1 SD (i.e., 33.7), the PT decreases by 0.413. The significant negative determinants of PT results according to the *β* values were FII, FV, FVII, and FIX. Interestingly, protein C was a significant positive determinant of PT. Meanwhile, aPTT was mainly dependent on FII, FIX, and FX.

In the TGA, the significant positive determinants of lag time were fibrinogen, protein C, protein S, and, to a lesser extent, FV with both 1 and 5 pM TF. With both 1 and 5 pM TF, FIX and protein C were the strongest positive and negative determinants of peak thrombin, respectively. With 5 pM TF, ETP was positively determined by FII and negatively determined by antithrombin and protein C; meanwhile, with 1 pM TF, ETP was positively determined by FIX and negatively determined by protein C alone. 

### 3.2. Hypercoagulability Expressed as ETP Ratio in Cirrhosis

**Figure 1:**
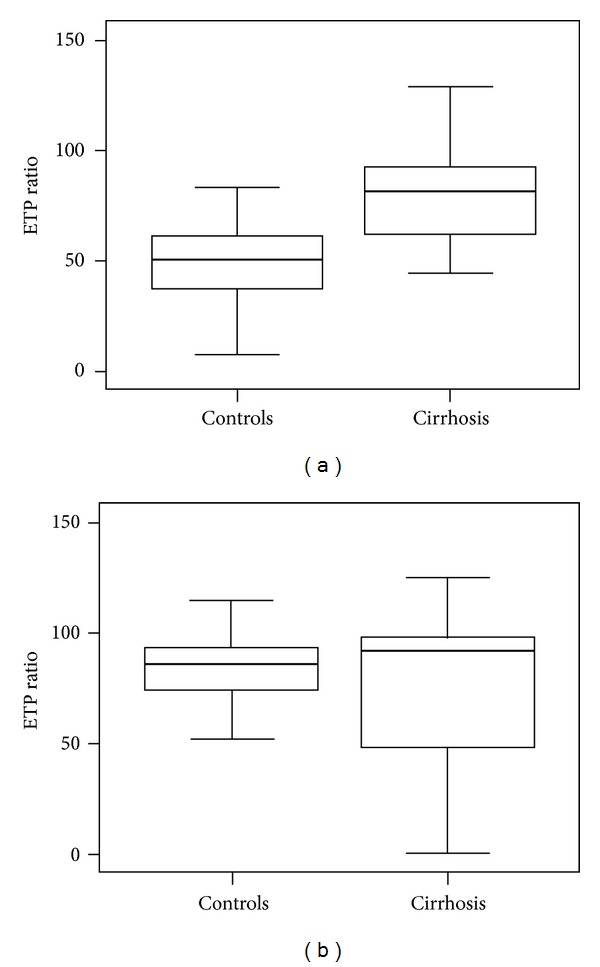
Endogenous thrombin potential (ETP) ratios of healthy controls (*n* = 73) and cirrhotic patients (*n* = 156). The ETP ratio was calculated by dividing “ETP with thrombomodulin (TM)” by “ETP without TM” multiplied by 100. The concentrations of TM were 2.5 nM for 1 pM tissue factor stimulation and 5 nM for 5 pM tissue factor stimulation. (a) With 1 pM tissue factor stimulation, the ETP ratio was significantly higher in the patients with cirrhosis (*P* < 0.001). (b) With 5 pM tissue factor stimulation, there was no significant difference in the ETP ratio between the controls and patients with cirrhosis.

The ETP ratio with 1 pM TF stimulation was significantly higher in patients than that in controls (Figure 1(a)), whereas there was no significant difference between groups with 5 pM TF (Figure 1(b)). 

**Figure 2:**
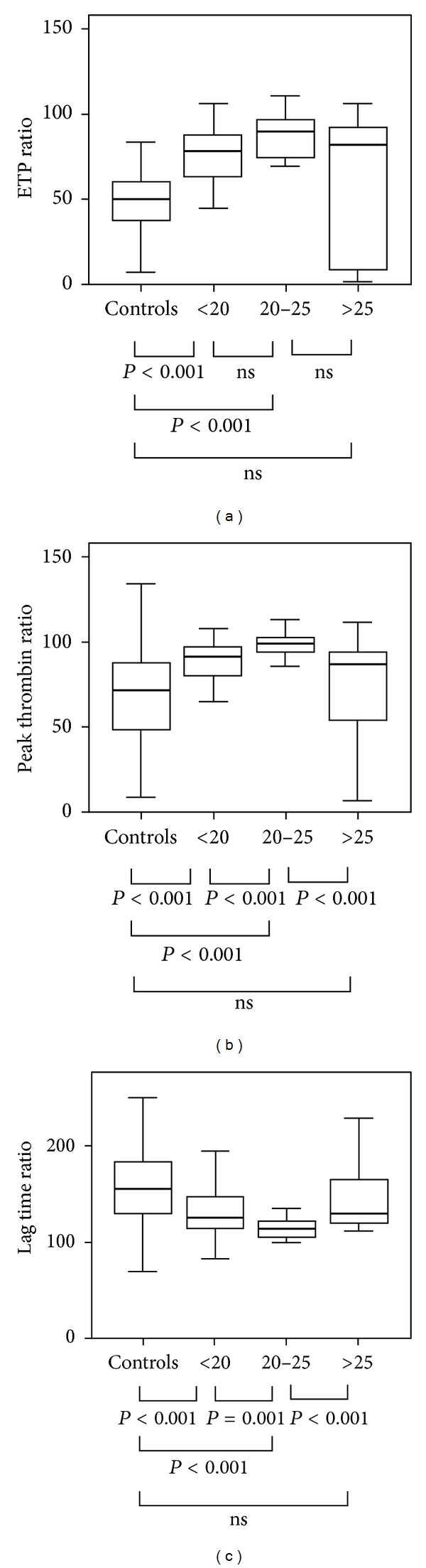
(a) The endogenous thrombin potential (ETP) ratio, (b) peak thrombin ratio, and (c) lag time ratio with 1 pM tissue factor stimulation according to the model of end-stage liver disease (MELD) scores of cirrhotic patients and controls.

When the patients were subdivided into 3 groups according to their model of end-stage liver disease (MELD) scores (i.e., low, middle, and high), the ETP ratio with 1 pM TF was significantly increased at both group with <20 of MELD and group with 20–25 of MELD, compared with that of controls. However, the ETP ratio of group with >25 of MELD was not significantly increased, compared with that of group with 20–25 of MELD (Figure 2(a)). A similar pattern was observed with peak thrombin ratio (Figure 2(b)). In contrast, the lag time ratio decreased gradually until the middle range of MELD scores (Figure 2(c)). 

## 4. Discussion

Patients with cirrhosis exhibit impaired coagulation function. These hemostatic changes can be measured using conventional global coagulation assays such as the PT and aPTT. However, recent reports suggest the TGA is superior for assessing coagulation status because it involves more physiological systems and measures total thrombin generation over time [5–7]. Although these global coagulation assays are available in clinical laboratories, there was no data thus far about the influence of pro- and anticoagulation factors on PT, aPTT, or TGA in cirrhosis. 

The results show that the levels of FII, FV, FVII, and FIX significantly influenced the PT. Because the liver synthesizes all coagulation factors except FVIII, PT prolongation is expected in cirrhosis. However, it is unknown whether anticoagulant proteins such as protein C and protein S, which decreased in cirrhosis, influence the PT. Interestingly, our study revealed that protein C levels influence the PT, indicating that protein C plays a role in PT prolongation via the inactivation of FV and FVIII in the PT system *in vitro*, similar to coagulation* in vivo*. To our knowledge, there has been no report about the effect of protein C on PT value. Since patients with cirrhosis exhibit decreased levels of both coagulation and anticoagulation factors, decreased levels of coagulation factors may prolong the PT, while decreased anticoagulation factors may shorten the PT. Therefore, PT prolongation may not be prominent in cirrhosis unlike changes in other liver function markers. Since PT is a component of the Child-Pugh score as well as the MELD score, which is the most commonly used marker to assess the severity of liver disease [9], it is important for physicians to understand that PT is dependent on protein C as well as coagulation factors. 

The aPTT was mainly dependent on FII, FIX, and FX levels. However, anticoagulation factors did not affect the aPTT. Therefore, the aPTT is not a useful test for detecting overall coagulation potential in cirrhosis. Another report states that the aPTT is not a good predictor of hemorrhage either [1].

In our TGA experiments, the lag time of the TGA was positively determined by fibrinogen and anticoagulants including protein C and protein S. It is reasonable that the increased protein C and proteinS levels prolong the lag time. The fibrinogen is thought to make the lag time reduced. However, our results show that fibrinogen level paradoxically affect the prolongation of lag time. Since fibrinogen is a well-known acute phase protein [10], this fibrinogen level may be increased in inflammatory status of cirrhotic patients. After all, the increased fibrinogen levels are accompanied by the reduced levels of other coagulation factors in cirrhotic patients. Therefore, the fibrinogen may reflect the other coagulation factors in statistical analysis. In other words, the increased fibrinogen seems to prolong the lag time on the specimen that had low levels of other coagulation factors. 

Peak thrombin was significantly dependent on FIX and protein C levels. Dielis et al. [11] reported the influence of coagulation factors on peak thrombin in healthy individuals, in which the main determinants of ETP at 1 pM TF were fibrinogen, FXII, tissue factor pathway inhibitor, and antithrombin. Because their study was based on data of healthy individuals, the determinants of ETP may be quite different from results of our cirrhotic population. In our results, ETP was highly dependent on protein C. Regarding the result, it is noteworthy that protein C levels consistently affected the 3 parameters of the TGA. Thus, the TGA appears to be sensitive to protein C levels. Therefore, the TGA is expected to be a good global assay for estimating overall coagulation activity, especially in clinical conditions with low protein C levels, such as cirrhosis and acquired or congenital protein C deficiency. 

Thrombotic events can paradoxically occur in cirrhotic patients even if clinically prolonged PT results are considered to suggest hemorrhagic tendency. Tridopi et al. [4] report that cirrhosis patients exhibit hypercoagulability according to the ETP ratio with 1 pM TF stimulation. In the present study, we investigated the ETP ratio with both 1 and 5 pM TF stimulations to evaluate cirrhotic hypercoagulability. As expected, the ETP ratio with 1 pM TF stimulation was higher in patients with cirrhosis than that in controls, whereas there was no difference between groups with 5 pM TF stimulation. The TF concentration used in the original test was 5 pM; meanwhile, the 1 pM TF concentration was used to increase sensitivity to FVIII, FIX, and FXI [12]. Since hypercoagulability in cirrhosis is considered to be due to increased FVIII and decreased protein C levels [13], the ETP ratio with 1 pM TF stimulation is thought to be higher in cirrhosis. On the contrary, the ETP ratio with 5 pM TF stimulation was not sensitive to FVIII concentration; therefore, it cannot be used to detect the hypercoagulability of cirrhosis. This finding suggests that the original TF concentration of 5 pM is not an appropriate stimulation for ETP to assess the hypercoagulability of cirrhotic patients. 

The ETP ratio represents enhanced resistance to the anticoagulant action of thrombomodulin. We demonstrated that the ETP ratio with 1 pM TF stimulation increased gradually with respect to the MELD score until a score of 25. However, the ETP ratio of the patients with MELD scores > 25 was not more than that in those with low MELD scores. A similar pattern was observed with the peak thrombin time, while the opposite pattern was observed with the lag time ratio. Further studies using larger patient groups with MELD scores > 25 are required to confirm whether this hypercoagulability is mainly confined to patients with low to middle MELD scores. 

A limitation of the present study is that contact activation may affect the TGA assay* in vitro* [14]. Avoiding contact inhibition in clinical practice is not feasible, because a special tube with a high cost and short shelf life must be used to inhibit contact activation. Moreover, the physiological roles of contact factors *in vivo* remain unclear. Therefore, the present results merely demonstrate the usefulness of TGA as a practical test in clinical laboratory settings. 

## 5. Conclusions

In conclusion, both coagulation and anticoagulation factors affect the results of global coagulation assays such as the PT and TGA. Of note, protein C levels strongly affect the PT and all parameters of the TGA. Patients with cirrhosis exhibit hypercoagulability in terms of their ETP ratio with 1 pM TF stimulation despite their prolonged PT. Although routine coagulation tests such as the PT and aPTT do not detect the thrombotic tendency in cirrhosis, the TGA can detect hypercoagulability in cirrhosis. It would be interesting to evaluate the global coagulation status using the newly devised TGA system in cirrhosis patients in the future.

## Figures and Tables

**Table 1:** Clinical and laboratory characteristics of the patients and controls.

		Cirrhotic patients				
	Controls (*n* = 73)	Total	MELD < 20	MELD 20–25	MELD > 25	**P value
		(*n* = 156)	(*n* = 78)	(*n* = 37)	(*n* = 41)	
Age (years)	54 (50–62.50)	57.5 (52–66.75)	55 (50–67)	59 (57–68.5)	57 (47–65)	0.050
Gender						
Male	43 (58.9%)	99 (63.5%)	52 (65.8%)	23 (62.2%)	25 (61.0%)	0.852
Female	30 (41.1%)	57 (36.5%)	27 (34.2%)	14 (37.8%)	16 (39.0%)	
Etiology of cirrhosis						
ALD		16 (10.3%)	6 (7.7%)	3 (8.1%)	7 (17.1%)	
HCV		4 (2.6%)	2 (2.6%)	0 (0.0%)	2 (22.0%)	
HBV		16 (10.3%)	13 (16.7%)	0 (0.0%)	3 (56.1%)	0.406
Cryptogenic		40 (25.6%)	26 (33.3%)	12 (32.4%)	2 (4.9%)	
HCV and HCC		20 (12.8%)	6 (7.7%)	7 (18.9%)	7 (0.0%)	
HBV and HCC		60 (38.5%)	25 (32.1%)	15 (40.5%)	20 (0.0%)	
Child-Pugh B		84 (53.8%)	65 (82.3%)	13 (35.1%)	6 (14.6%)	<0.001
Child-Pugh C		72 (46.2%)	13 (16.5%)	24 (64.9%)	35 (85.4%)	<0.001
AST (IU/L)	25.0 (19.0–30.0)	49.5* (31.3–95.3)	37.0 (28.0–63.0)	47.0 (29.0–80.5)	100.0 (48.0–295.0)	<0.001
ALT (IU/L)	24.0 (19.0–34.0)	32.5* (20.0–68.8)	28.0 (19.0–53.0)	23.0 (18.0–39.0)	64.0 (26.5–107.5)	0.001
ALP (IU/L)	50.0 (43.0–61.0)	99.0* (70.3–153.0)	92.0 (67.0–139.0)	97.0 (69.0–121.5)	122.0 (90.5–254.0)	0.004
WBC (×10^9^/L)	4.95 (4.28–5.85)	5.22 (3.60–12.10)	5.30 (3.49–9.20)	4.24 (3.27–4.59)	12.09 (5.20–18.84)	<0.001
Platelet (×10^9^/L)	233 (203–282)	154*(48–128)	90 (61–187)	59 (41–87)	64 (44–88)	<0.001
Bilirubin (mg/dL)	1.0 (0.8–1.3)	3.2* (1.2–14.4)	1.7 (0.8–3.0)	5.7 (1.6–14.0)	18.0 (14.3–28.1)	<0.001
Albumin (g/L)	4.2 (4.0–4.3)	2.9* (2.5–3.3)	3.0 (2.9–3.5)	2.5 (2.4–3.3)	2.6 (2.4–2.9)	<0.001
Creatinine (mg/dL)	0.94 (0.79–1.02)	0.92 (0.67–1.26)	0.77 (0.62–0.95)	0.94 (0.60–1.40)	1.27 (0.96–2.24)	<0.001
PT (s)	10.9 (10.5–11.4)	23.1* (13.7–27.0)	66.0 (38.0–83.0)	32.0 (28.0–38.0)	35.0 (26.0–38.5)	<0.001
aPTT (s)	30.5 (29.2–33.0)	39.5* (33.4–48.3)	34.2 (30.3–44.4)	41.3 (38.5–57.2)	45.5 (39.2–64.3)	<0.001
Antithrombin (%)	107.7 (97.7–113.7)	47.6* (21.3–68.5)	59.9 (51.6–92.2)	24.3 (15.0–47.0)	21.1 (12.0–28.0)	<0.001
Protein C (%)	113.6 (103.7–122.0)	32.8* (13.6–53.1)	48.7 (36.0–65.5)	13.9 (10.8–37.2)	14.6 (11.4–20.3)	<0.001
Protein S (%)	93.7 (81.7–102.3)	54.5* (43.2–68.6)	54.9 (44.3–75.4)	49.2 (39.6–59.8)	55.9 (44.5–73.8)	0.213

*The Mann-Whitney *U* test was used between controls and total patients of cirrhosis.

**The Kruskal-Wallis test was used for continuous variables and *χ* ^2^ test for discrete variables among three cirrhosis subgroups.

Data are expressed as the median (interquartile range) for continuous variables and number (percentage) for categorical variables unless indicated otherwise.

ALD: alcoholic liver disease; HCV: hepatitis C virus; HBV: hepatitis B virus; PBC: primary biliary cirrhosis; PSC: primary sclerosing cholangitis; AST: aspartate aminotransferase; ALT: alanine aminotransferase; ALP: alkaline phosphatase; WBC: white blood cell count; PLT: platelet count; INR: international normalized ratio; PT: prothrombin time; aPTT: activated partial thromboplastin time; MELD: model of end-stage liver disease.

**Table 2:** Multivariate regression analysis of the determinants of global coagulation tests in cirrhosis (*n* = 156).

		PT	aPTT	Lag time		Peak thrombin		ETP	
	SD			5 pM TF	1 pM TF	5 pM TF	1 pM TF	5 pM TF	1 pM TF
		*β*	*β*	*β*	*β*	*β*	*β*	*β*	*β*
(Adjusted *R* ^2^)		0.779	0.633	0.491	0.394	0.509	0.348	0.465	0.265
Fibrinogen	111.9	0.092	−0.005	0.640^†^	0.602^†^	0.032	−0.219*	−0.044	−0.200
FII	33.7	−0.413*	−0.659*	−0.311	−0.434	0.455*	0.005	1.339^†^	0.427
FV	22.6	−0.146*	0.027	0.166*	0.106	0.123	0.015	0.000	−0.081
FVII	39.1	−0.297^†^	0.089	−0.241	−0.004	0.051	0.127	−0.233	0.098
FVIII	60.6	0.038	−0.179	0.029	0.102	−0.047	−0.083	0.133	−0.097
FIX	35.3	−0.291*	−0.680^†^	−0.145	−0.281	0.614^†^	0.990^†^	0.183	0.717^†^
FX	34.4	−0.150	0.507*	−0.210	−0.216	0.077	0.265	0.233	0.119
Antithrombin	53.6	0.041	−0.088	0.023	0.026	0.001	−0.057	−0.227*	−0.158
Protein C	44.4	0.301*	−0.025	0.364*	0.466*	−0.657^†^	−0.846^†^	−0.796^†^	−1.093^†^
Protein S	25.8	0.019	−0.041	0.281^†^	0.256*	0.019	−0.121	−0.099	−0.027

Data are expressed as standardized regression coefficients (*β*). *P < 0.05; ^†^ *P* < 0.001.

F: factor; PT: prothrombin time; aPTT: activated partial thromboplastin time; ETP: endogenous thrombin potential; TF: tissue factor.
